# Contralateral approach using microscope and tubular retractor system for ipsilateral decompression of lumbar degenerative lateral recess stenosis associated with narrow spinal canal

**DOI:** 10.3389/fneur.2024.1387801

**Published:** 2024-04-17

**Authors:** Longfei Shu, Qingchun Mu, Feihu Dai, Wei Zhao, Madiha Zahra Syeda, Yuhai Wang

**Affiliations:** ^1^Department of Neurosurgery, Wuxi Clinical College of Anhui Medical University, 904th Hospital of Joint Logistic Support Force of PLA, Wuxi, China; ^2^Department of Neurosurgery, Second Affiliated Hospital of Soochow University, Suzhou, China; ^3^Keenan Research Centre for Biomedical Science, St. Michael's Hospital, Toronto, ON, Canada; ^4^Faculty of Medicine, Institute of Medical Sciences, University of Toronto, Toronto, ON, Canada

**Keywords:** contralateral approach, laminectomy alone, lateral recesses stenosis, minimally invasive, microscope

## Abstract

**Objective:**

To summarize the clinical effect of a single-center retrospective analysis of the contralateral approach with a microscope and tubular retractor system for ipsilateral decompression in patients with lumbar lateral recess stenosis and a narrow spinal canal.

**Methods:**

A total of 25 patients who underwent ipsilateral decompression surgery via a contralateral approach with microscope and tubular retractor system, performed by one surgeon at a single center were retrospectively examined. The width of the lamina fenestration was compared with the preoperative distance from the root of the spinous process to the dorsal articular facet, the bilateral articular facet change in the suprapedicle notch section on CT scan, and with the changes in transverse and sagittal diameters of the canal area on MRI. Clinical efficacy was assessed using the Japanese Orthopedic Association (JOA), Visual Analog Scale (VAS), and Oswestry Disability Index (ODI) scores.

**Results:**

In total, 25 patients were treated and the mean intraoperative time was 82.04 ± 12.48 min. There was no nerve injury, cerebrospinal fluid leakage, and infection complications. The postoperative CT revealed that the width of the contralateral laminar fenestration was less than the distance from the root of the spinous process to the dorsal articular facet. The residual widths of the ipsilateral articular facet and contralateral articular facet were greater than 2/3 of the preoperative articular facet width. The transverse and sagittal diameter of canal were significantly increased. The mean follow-up period was 12–16 months, and no recurrence or reoperation incidence were found at the last follow-up. When compared to pre-surgery, the ODI, VAS, and JOA scores were significantly improved after surgery (*p* < 0.05).

**Conclusion:**

Based on our single-center retrospective observation of 25 cases and combined with previous literature, the contralateral approach with a microscope and tubular retractor system for ipsilateral decompression in patients with lumbar lateral recess stenosis and a narrow spinal canal can reduce damage to the articular processes, and probably more conducive to the postoperative stability of the lumbar spine. This was a single center retrospective analysis with a small sample size and lacked randomized controlled trials (RCTs). However, larger-scale, multicenter RTCs are required for additional validation.

## Introduction

Currently, lumbar spinal stenosis is the most prevalent spinal disease affecting adults over 65 years old (1–3). Based on the location of the stenotic pathology, lumbar spinal stenosis can be anatomically classified into three types: central stenosis, lateral recess stenosis, and foraminal stenosis (4). Degenerative lateral recess stenosis is usually caused by hyperplasia of the articular facets, osteophyte formation, hypertrophied ligamentum flavum, or disc herniation (5). A hypertrophied ligamentum flavum and hyperplasia of the articular facet on the dorsal side are the main causes of spinal canal and nerve root compression (6).

The aging process contributes to an increase in the number of geriatric patients with degenerative lumbar lateral recess stenos is worldwide. These patients usually have severe symptoms, protracted illness, and poor overall health. It is important to take into account the ease, safety, and efficacy of the treatments that can be performed to alleviate pain and discomfort and improve the quality of life (7, 8). Additionally, conservative treatment yields no discernible results. The traditional posterior surgical approach typically entails decompression with foraminotomy and facetectomy (9, 10, 11). which enlarges the nerve root canal but also increases intraoperative tissue destruction, blood loss, and postoperative lumbar instability, and can be performed with lumbar fusion (12, 13).

The development of tubular retractors in 1997 and the subsequent development of endoscopic techniques have led to a pattern shift from open to minimally invasive surgeries (14, 15, 16). In 2002, Palmer et al. reported the feasibility and surgical efficacy of unilateral approach bilateral decompression (ULBD) and the use of a tubular retractor system in patients with LSS and degree I spondylolisthesis (17, 18). Further, patients who present with unilateral lateral recess or foraminal stenosis are frequently treated with an ipsilateral microscope or endoscopic approach (19, 20).

**Figure 1:**
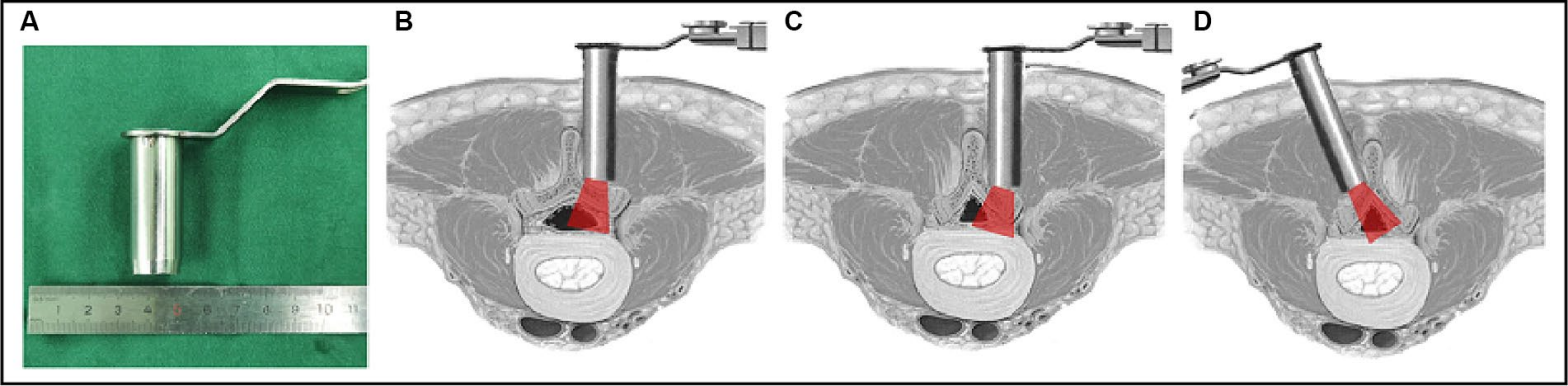
Illustration of the tube in a vertical and medially angled position to access the contralateral side. **(A)** Diameter of the tube; **(B)** a normal vertebral lamina with ipsilateral decompression through the symptomatic ipsilateral approach; **(C)** a narrow vertebral lamina with ipsilateral decompression through the symptomatic ipsilateral approach; **(D)** a narrow vertebral lamina with ipsilateral decompression through the symptomatic contralateral approach.

The diameter of the working tube in our center was 1.6 cm (Figure 1A), which is smaller than the width of most lamina. The ipsilateral microscopic approach was used more frequently to perform the lateral recess decompression without excessively grinding the articular joints (Figure 1B); however, in some cases, the lamina’s width combined with wide spinous processes or a spinal canal that is developmentally narrower than the tubular diameter may cause problems. Surgeons using the ipsilateral microscope approach are likely to over-resect the inferior articular process, which can easily lead to excessive breakthrough of the articular joint, raising the risk of postoperative instability (Figure 1C). In contrast, the contralateral approach does not require excessive grinding of the articular joint to expose the lateral recess, and utilizes the inclination angle of the tube to complete symptomatic nerve root decompression (Figure 1D). The KOIKUTA study also showed that articular processes on the ipsilateral side suffered more damage than contralateral side during unilateral bilateral decompression (21). Alimi et al. showed that contralateral foraminotomy is a preferable option for decompression on the opposite side (22). Concordontaly, the percutaneous endoscopic contralateral technique, which has also been shown to lessen injury to the lumbar articular processes on the approach side, was employed by Hyeung Sung Kim to treat lumbar spine lateral recess stenosis (23).

Here, we reported the lamina fenestration, bilateral articular facet changes, and radiological outcomes of the standalone contralateral approach for ipsilateral lateral recess decompression, using a microscope and tubular retractor system in patients with a bilateral distance of less than 1.6 cm from the root of the spinous process to the dorsal articular facet in the suprapedicle notch section CT scan. We have discussed and compared our findings with the current literature on the contralateral approach.

## Materials and methods

### Characteristics of patients and study design

A retrospective study of 317 consecutive patients with lumbar spinal stenosis was conducted from September-2019 to December-2022 at the 904th Hospital of Joint Logistic Support Force of the PLA. Of these, 25 patients presented with degenerative ipsilateral recess stenosis associated with a narrow spinal canal and underwent decompression via a contralateral approach with a microscope and tubular retractor system operated by one senior surgeon at a single center.

### Inclusion/exclusion criteria

All patients underwent anterior and lateral lumbar radiography, computed tomography (CT), and magnetic resonance imaging (MRI) before surgery. Every patient satisfied the following inclusion and exclusion criteria and had a diagnosis of lateral recess stenosis. Inclusion criteria: (1) aged 45 years or older; (2) width of the bilateral vertebral plate from the root of the spinous process to the dorsal articular facet in the suprapedicle notch section CT scan less than 1.6 cm; (3) lumbar spondylolisthesis less than or equal to grade I; and (4) no significant effect of conservative treatment for 6 months. Exclusion criteria: (1) acute lumbar disc herniation resulting in lateral recess stenosis; (2) a distance of more than 1.6 cm from the root of the spinous facet to the articular surface on CT scan of the suprapedicle notch section; (3) bilateral lateral recess stenosis; (4) a lumbar spondylolisthesis exceeding grade I; (5) a history of lumbar spine decompression surgery, spinal tumors, or spinal tuberculosis; and (6) an unwillingness to adhere to follow-up agreements.

### Radiological methods

**Figure 2:**
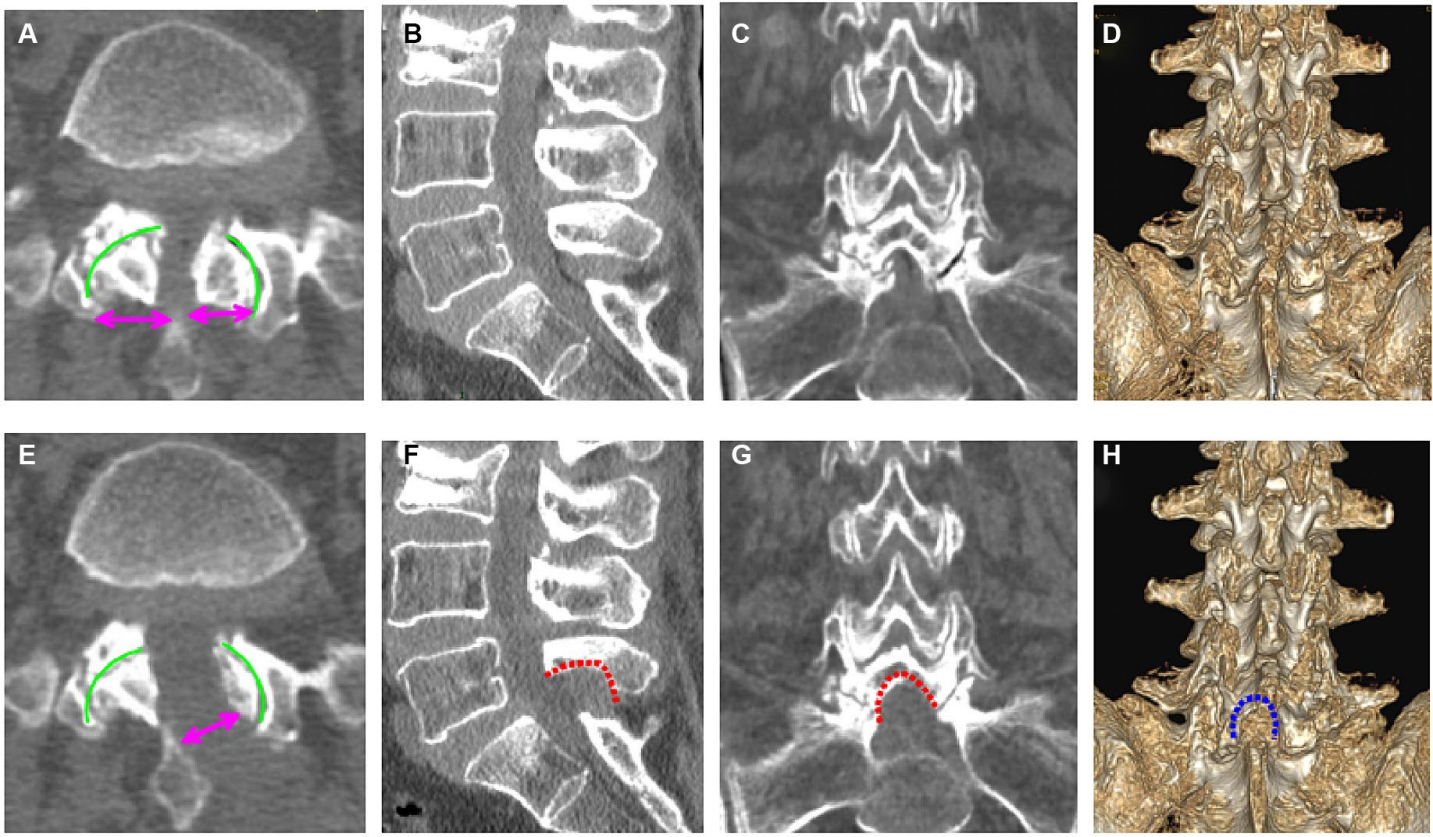
Pre- **(A–D)** and postoperative **(E–H)** computed tomography (CT) images of the lumbar spine (a 69-year-old woman). **(A)** Preoperative axial CT image of L5/S1; **(B)** preoperative sagittal CT image of L5/S1; **(C)** preoperative coronal CT image of L5/S1; **(D)** preoperative three-dimensional CT image of L5/S1; **(E)** postoperative axial CT image of L5/S1; **(F)** postoperative sagittal CT image of L5/S1; **(G)** postoperative coronal CT image of L5/S1; **(H)** three-dimensional CT image of L5/S1.

**Figure 3:**
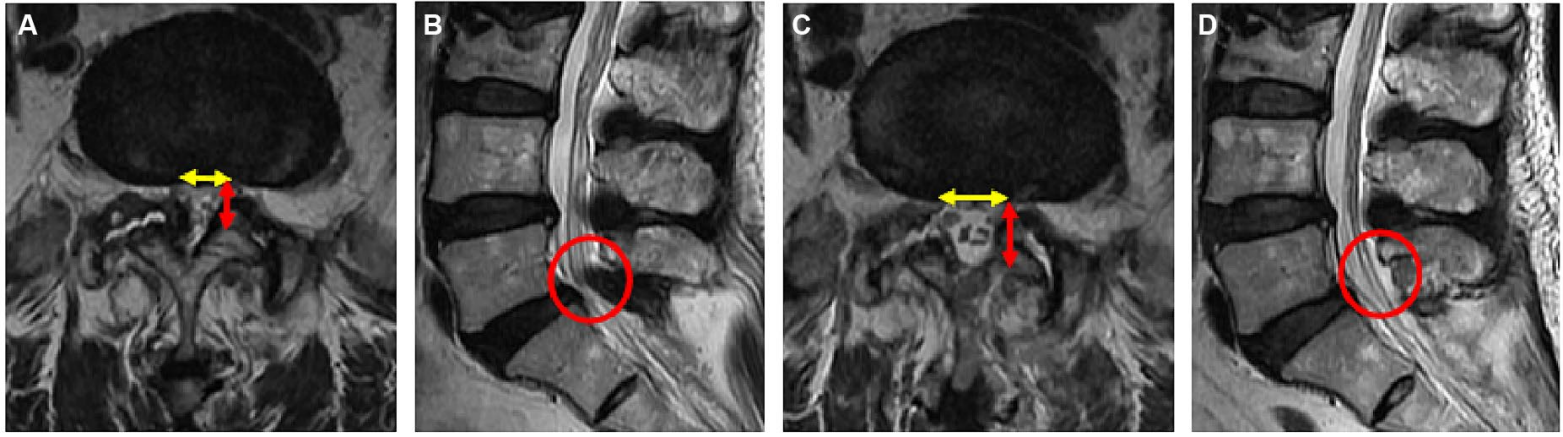
Pre- **(A,B)** and postoperative **(C,D)** magnetic resonance imaging (MRI) of the lumbar spine in a 69-year-old female patient. **(A)** Preoperative axial MR image of L5/S1; **(B)** preoperative sagittal MR image of L5/S1; **(C)** postoperative axial MR image of L5/S1; **(D)** postoperative sagittal MR image of L5/S1.

Using the CareStream Pacs imaging system (OneX, Canada), two senior doctors measured the distance of the bilateral lamina from the spinous process root to the bilateral dorsal articular facet based on the CT image of the suprapedicular notch section as well as the width of the bilateral articular facet (Figures 2A,E). The transverse and sagittal diameters of the dural sac in the axial plane were measured via MRI (Figures 3A,C). All the indicators were measured twice, and the average value was calculated.

### Surgical methods

**Figure 4:**
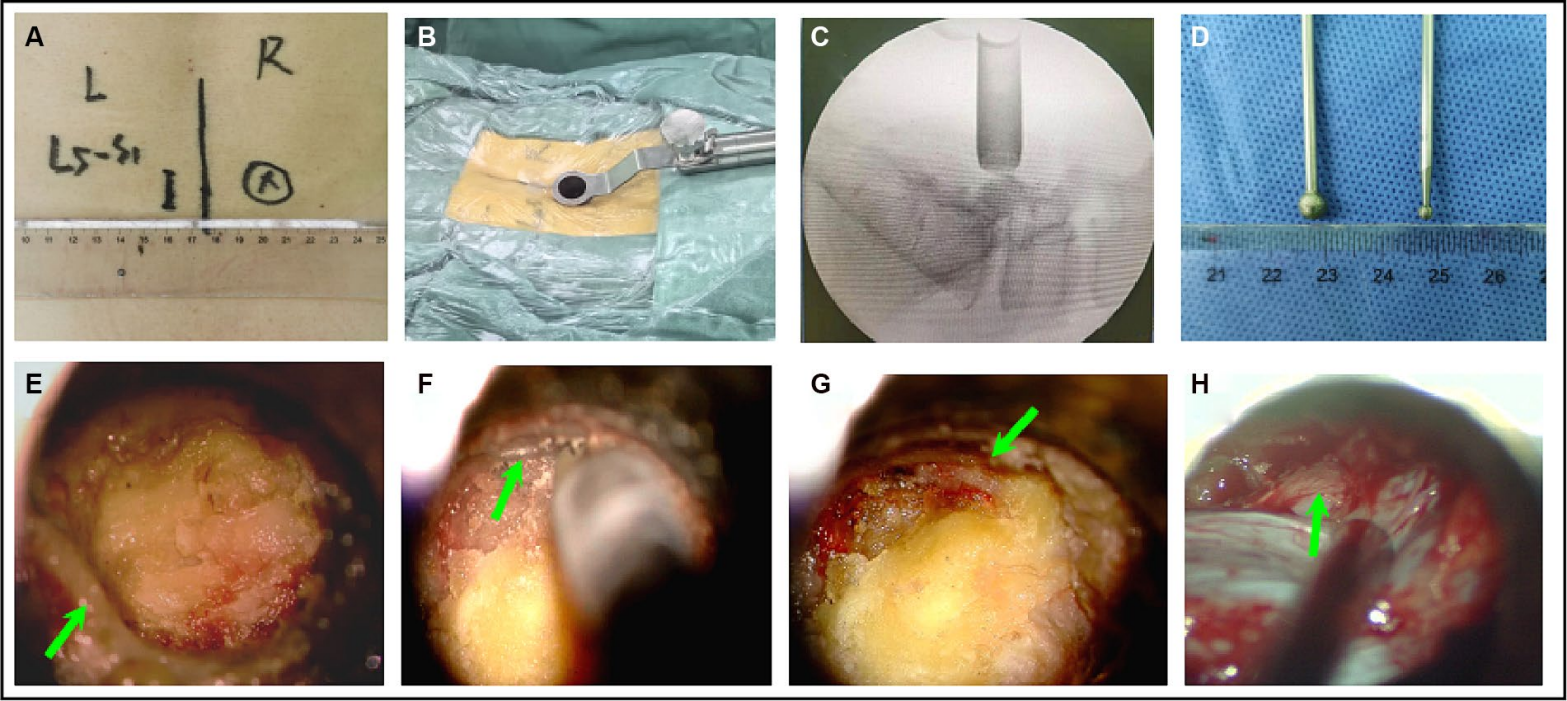
The surgical procedure for microscopic tubular reconstruction: A working tube for microchannel decompression was established **(A–C)**. **(D)** The style of the high-speed drill. The left part of the inferior articular process was removed to expose the ligamentum flavum and contralateral interlaminar region **(E,F)**. Ipsilateral osteophytes **(G)**. The ligamentum flavum and the ipsilateral nerve root were removed **(H)**.

(1) Surgical position, anesthesia and body surface positioning: Under general anesthesia and in the prone position with neuroelectrophysiological monitoring. The target level was verified using C-arm guidance, and a 1.8–2.0 cm paramedian skin incision was made overlying the target level approximately 1.5–2 cm lateral to the midline on the patient’s symptomatic contralateral side (Figure 4A). (2) Establishment of the spinal tubular working channel: The skin, fascia, and muscle were sequentially dilated, after which we inserted a 16-mm modified mini-retractor microscopic tube of the shortest length, usually 50 or 70 mm, that would allow adequate depth of access (Figure 4B). The surgical level was verified by C-arm guidance (Figure 4C). (3) Contralateral lamina fenestration: An operating microscope (Leica OH4, Germany) was used to view the fenestration into the field, and the paraspinal muscles were removed by an electrocautery from their bony attachments on the spinous process and lamina to expose the bony details. The inferior edge of the lamina was defined, and a hemilaminotomy was performed with a width diameter of 5 mm high-speed drill (Figure 4D) extending cranially above the attachment point of the ligamentum flavum on the inferior lamina surface, caudally on the superior lamina surface, and on the medial margin of the articular process. (4) The inner edge of the lamina was grounded on the ipsilateral side: The working tube was then angled medially to expose the anterior aspect of the spinous process, which was then removed utilizing a width diameter of 2 mm high-speed drill by microscopic angulation (Figure 4D), adhering to the inner edge of the vertebral plate on the symptomatic side with trumpeted decompression (Figures 4E,F). The inferior articular process of the upper hemilaminectomy was removed, and the inner edge of the articular facet of the articular process was revealed, than the superior articular process led to ipsilateral recess stenosis was exposed. During the use of high-speed drill, ligandum flavum removal should not be performed until sufficient medial facetectomy is performed. (5) Removed the ligamentum flavum and decompressed the nerve root: The ligamentum flavum and ipsilateral facet could be resected using Kerrison punches (Figure 4G). The dural sac was separated to the lamina fenestration side, and the exiting nerve root was completely decompressed to the internal edge of the pedicle side under direct visualization. The ipsilateral nerve root were then completely decompressed (Figure 4H). (6) Final step: The surgical cavity was rinsed with hydrogen peroxide, diluted iodine solution, and physiological saline sequentially; the tubes were removed; the paraspinal muscles were repositioned; the muscle membrane, subcutaneous tissue, and skin were sutured sequentially; and suction drains were routinely placed.

### Outcome assessment

Prior to surgery, the Japanese Orthopedic Association (JOA) score, the Disability Scale (Oswestry Disability Index [ODI]), and the Scale for Leg Pain (visual analog scale [VAS]) were used to assess the functional limits and symptoms of the patients. All the assessments were repeated at 7 days, 1 month, 3 months, 6 months and 12 months post-surgery. Postoperative CT scan was used to evaluate the width of the contralateral vertebral lamina fenestration and the remaining width of the bilateral articular facet in all patients. Similarly, postoperative MRI was used to determine the changes in the transverse and sagittal diameters of the dural sac.

### Statistical analysis

Statistical analysis was performed using SPSS (version 19.0; SPSS, Inc., Chicago, IL, United States). Continuous variable data are presented as the mean ± standard deviation (SD), with discontinuous variables as percentages. For quantitative data, a *t*-test was used when the data conformed to a normal distribution. For the quantitative data, *t*-test was selected when it conforms to normal distribution. The paired *t*-test was used for the quantitative data with equal variance assumed between the two groups, and the separate variance estimation *t*-test was used for the quantitative data with equal variance not assumed. The Chi-square test was used to assess the qualitative data between the two groups. Significance was set at *p* < 0.05.

## Results

### Baseline clinical and demographic characteristics

A total of 25 patients with a mean age of 65.16 ± 7.43 years (range 54–82 years) were included in the study, 13 of whom were male (52%) and 12 of whom were female (48%). The symptoms persisted for 6 to 48 months. The lateral recess stenosis levels were as follows: L3-4 (in 8 patients, 32%), L4-5 (in 10 patients, 40%), and L5-S1 (in 7 patients, 28%). Twenty (80%) patients showed decreased sensation in clinical symptoms, 23 (92%) had a positive lower limb nerve tension test, and 18 (76%) had neurogenic claudication. The surgical duration was 82.04 ± 12.48 min (60–100 min), and intravenous cefazolin was administered both pre- and post-surgery. An 80 mg dose of depomedrol was given for 2 days to prevent nerve root edema. An exercise program was started after 1 day to strengthen the paravertebral muscles, and patients were allowed to leave bed with a lumbosacral corset after 2 days and discharged within 1 week. The baseline data are presented in Table 1.

**Table 1:** Baseline characteristics, surgery time and lamina width change values.

Level	n	Age (Mean + SD)	Male n (%)	DS n (%)	PNTT n (%)	NC n (%)	ST min (Mean ± SD)	Lamina width (Mean ± SD)	LF mm (Mean ± SD)
								Ipsilateral contral	
L3-L4	8	63.98 ± 8.90	5(62.5)	7(87.5)	8(100)	6(75)	85.75 ± 10.99	12.38 ± 1.51 12.38 ± 1.41	8.31 ± 0.65*
L4-L5	10	66.60 ± 6.57	5(50)	8(80)	9(90)	7(70)	77.50 ± 14.03	13.20 ± 1.63 13.22 ± 1.92	9.33 ± 1.42*
L5-S1	7	64.57 ± 7.59	3(42.8)	5(71.4)	6(85.7)	5(71.4)	84.29 ± 11.34	14.20 ± 1.83 14.14 ± 1.89	9.28 ± 0.75*
Total	25	65.16 ± 7.43	13(52)	20(80)	23(92)	18(76)	82.04 ± 12.48	13.46 ± 1.75 13.54 ± 1.69	9.23 ± 1.32*

DS, decreased sensation; PNTT, positive nerve tension test; IC, neurogenic claudication; ST, surgery time; LF, lamina fenestration. *P* < 0.001 versus preoperative values.

**Table 2:** Results of articular process measurements with pre- and postsurgical values.

Level	n	Ipsilateral Mean ± SD				Contralateral Mean ± SD			
		Preop×2/3	Postop	*t-*value	*p*-value	Preop×2/3	Postop	*t*-value	*P*-value
L3-L4	8	12.67 ± 1.51	16.43 ± 1.72	8.74	<0.001	13.01 ± 1.85	16.53 ± 1.63	10.09	<0.001
L4-L5	10	12.17 ± 1.69	16.71 ± 2.13	5.32	<0.001	12.21 ± 1.21	14.89 ± 1.75	4.091	0.001
L5-S1	7	11.47 ± 1.92	14.30 ± 1.27	4.77	<0.001	11.49 ± 1.76	13.87 ± 2.23	5.65	<0.001
Total	25	12.13 ± 2.21	15.92 ± 3.88	8.46	<0.001	12.25 ± 2.8	15.18 ± 2.64	7.40	<0.001

Preop, preoperative; Postop, postoperative.

### Complications

There were no accidental nerve injuries, CSF leakage, or infections. Three patients experienced aggravated symptoms of lower limb numbness due to nerve root edema after surgery and recovered 1 week later of receiving hyperbaric oxygen and 80 mg of depomedrol treatment.

### Radiological results

The radiological results are shown in Tables 1–3. Postoperative CT (Figures 2B–H) and MRI (Figures 3B–D) scanning demonstrated adequate decompression and no instability in all patients, and the contralateral lamina fenestration width was 9.23 ± 1.32 mm, which was less than the distance between the spinous process to the dorsal articular facet (13.46 ± 1.75 mm) (*p* < 0.001). The residual widths of the ipsilateral articular facet (15.92 ± 3.8 mm) and contralateral articular facet (15.18 ± 2.64 mm) were greater than 2/3 of their preoperative widths (12.13 ± 2.21 mm and 12.25 ± 2.8 mm, respectively) (both *p* < 0.001). The canal area significantly increased from 8.29 ± 1.39 to 13.93 ± 1.82 mm (*p* < 0.001) in transverse diameter and from 9.27 ± 1.55 mm to 13.50 ± 2.40 mm in sagittal diameter (*p* < 0.001).

**Table 3:** Dural sac results of nuclear magnetic resonance measurements before and after surgery.

Level	n	Transverse diameter Mean ± SD				Sagittal diameter Mean ± SD			
		Preop	Postop	*t*-value	*p-*valu*e*	Preop	Postop	*t*-value	*p*-value
L3-L4	8	8.37 ± 0.96	14.26 ± 2.02	7.45	<0.001	8.87 ± 1.64	13.51 ± 2.82	4.01	0.001
L4-L5	10	7.54 ± 1.61	14.27 ± 1.87	9.64	<0.001	9.57 ± 1.69	14.76 ± 2.12	6.05	<0.001
L5-S1	7	9.27 ± 0.86	13.07 ± 1.43	6.02	<0.001	9.30 ± 1.35	11.70 ± 1.81	4.04	<0.001
Total	25	8.29 ± 1.39	13.93 ± 1.82	12.31	<0.001	9.27 ± 1.55	13.50 ± 2.40	7.40	0.002

Preop, preoperative; Postop, postoperative.

### Clinical outcomes

Clinical outcomes throughout the follow-up period are shown in Table 4. The mean follow-up time was 14.1 months (12–16 months). Routine radiological investigations were performed at the indicated time intervals, and follow-up data were obtained from the VAS score, ODI, and JOA score for all 25 patients. Among the 20 patients with preoperative numbness, 12 improved within 1 month, and 5 showed improvement within 6 months after surgery; however, there was no significant symptom relief in the remaining 3 patients. Prior to surgery, 23 patients had radiating lower limb pain, 16 of them experienced pain relief 1 day after surgery, and the remaining 7 patients experienced pain relief 7 days after the surgery. One month following surgery, all 18 patients with neurogenic claudication fully recovered, and no patient required reoperation for residual or recurrent spinal stenosis at the same segment(s) at the 12th–16th months period. All the patients did not develop postoperative instability, and required instrumentation-assisted secondary fusion (Table 4).

**Table 4:** Comparisons of VAS, JOA, and ODI scores before and after surgery.

Time	VAS leg pain scores	JOA	ODI (%)
Preop	6.87 ± 0.96	13.49 ± 3.50	69.22 ± 13.69
1w postop	3.91 ± 0.90*	20.09 ± 2.82*	29.00 ± 7.35*
1mo postop	2.22 ± 0.60*	23.17 ± 2.15*	22.04 ± 6.08*
3mo postop	1.44 ± 0.51*	25.09 ± 2.26*	17.57 ± 4.41*
6mo postop	0.87 ± 0.63*	26.26 ± 1.69*	14.78 ± 3.73*
12mo postop	0.48 ± 0.25*	27.22 ± 1.35*	12.78 ± 2.18*

VAS, visual analog scale; JOA, Japanese Orthopedic Association; ODI, Oswestry Disability Index; Preop, preoperative; Postop, postoperative; d, day; w, week; mo, month/month. *P < 0.05 versus preoperative values.

## Discussion

Lumbar spinal stenosis can be subdivided into three categories according to pathological location: central stenosis, lateral recess stenosis, and foraminal stenosis (4). Lateral recess stenosis is commonly associated with neurogenic claudication, radiculopathy pain, and motor and sensory deficits, which can lead to various pathologies of spinal compression, including spondylisthesis, osteophytes, disc herniation, and ligamentum flavum hypertrophy (5). The main reasons for degenerative lateral recess stenosis are often dorsal ligamentum flavum hypertrophy and osteophyte compression (6). When conservative therapy fails, surgical treatment is usually necessary for symptomatic lateral recess stenosis. The preferred intervention for treatment is to relieve nerve root canal compression. In most centers, the conventional ipsilateral midline approach—which incorporates several decompression techniques such laminectomy, foraminotomy, and laminotomy remains an efficacious intervention strategy. However, wide excision of facet joints to access the foramen can cause instability (24). Moreover, lumbar fusion and fixation is usually needed for the repair of spinal stability. The main goal of this study was to describe the use of the contralateral minimally invasive approach as an effective method for treating ipsilateral recess stenosis, while protecting the bilateral articular joint.

Patients who present with a unilateral lateral recess are frequently treated using an ipsilateral approach (19, 20). When decompressing the ipsilateral nerve root, it is necessary to grind out too many articular processes on the ipsilateral recess side, which can easily lead to excessive removal of the articular joint and impair the stability of the posterior spine column. Preservation of facet joints is the very important in successful outcome following micro-decompression surgery, especially in patients with very narrow lamina (21, 25, 26). The concept of a contralateral approach was briefly described by Wiltse and Spencer in 1988 as a part of an open lumbar spine approach (9). In 2002, Palmer et al. reported the feasibility and surgery-related efficacy of unilateral bilateral decompression and the utilization of a tubular retractor system in patients with LSS (17, 18) Post-surgery imaging scans reveal that the contralateral approach is more likely to result in entry into the lateral recess and intervertebral foramen space. Myung-Hoon also demonstrated good articular protection when treating lumbar spinal central stenosis with a bilateral contralateral approach (27). Ikuta et al. reported that the reduction in facet size of 22.6% and fracture of the inferior facet is up to 6% of patients with microendoscopic posterior decompress techniques (21). Matsumura et al. reported that patients whose with degenerative stenosis is more common in the lateral recess region (28). During ipsilateral decompression in this patients, the damage to facet joint would increase deformation and may lead to instability, while a contralateral approach, the facets are better preserved and thus the fusion and fixation can be avoided in such patients. Concordontaly, Alimi et al. reported less articular joint destruction and greater protection on the approach side when using a contralateral approach to treat lumbar lateral recess and intervertebral foramen stenosis via a microscope and tubular system (22).

Hamasaki et al. (29) observed biomechanical changes in the spine in fresh degenerative spinal specimens using stepwise resection of the medial aspect of the articular processes. When more than one-third of the lower edge of the bilateral upper vertebral lamina combined with the medial aspect of the articular processes was removed, both spinal flexion and rotation were affected. Ahuja et al. (30) used the finite element model to study lumbar spinal stability in young people and showed that when the facet joint was removed by more than 30%, the biomechanics of the spine changed. In our treatment of patients with partial lumbar spinal stenosis at a single-center, we discovered that vertebral lamina’s width was smaller than the inner diameter of the 1.6 cm channel. To achieve ipsilateral nerve root decompression, it is necessary to remove more than one-third of the lateral articular joints. Since the narrow spinal canal allows for the easiest access to the lateral recess space, we contend that degenerative lateral recess stenosis is related to it in this study. With a contralateral approach, the ipsilateral lateral recesses and central canal can all be accessed and decompressed with a single incision, preserving mechanical stability. Our results showed that there was less damage to the bilateral articular joints, and the remaining articular facet was greater than 2/3 of its preoperative value. Since the extent of articular facet removal in the contralateral and ipsilateral approaches is equal to or less than that described in biomechanical studies, the lumbar spine stability is presumably maintained. The clinical results of our study suggest that at the mid-term follow-up, the procedure does not appear to increase the risk of fusion due to instability.

In this study, a high-speed drill with a diameter of 5 mm was used for lamina fenestration to improve the speed of removal of the vertebral plate on the fenestration side. To avoid excessive removal of the lamina and reduce ligamentum flavum compression on the dural sac, a 2 mm diameter high-speed drill was used to adhere to the inner edge of the vertebral plate on the symptomatic side. In comparison with the over-the-top decompression technique (31), also referred to as lumbar endoscopic unilateral laminotomy for bilateral decompression, we can be more efficient grinding drills and operated with bimanualness. In addition, the over-the-top technique needs to be highlighted that performing a full-endoscopic decompression is a complex technique which requires advanced endoscopic skills and should only be considered for surgeons that are already familiar with this technique. The splitting technique allows for a satisfying central decompression, with minimal sparing the facet joints and the posterior neural arch, but it is far laterally to reach the foramina and which can be insufficient decompression of lateral recess (32, 33).

Most patients had successful surgical results without any nerve damage or post-operative cerebrospinal fluid leakage (34). We exposed the whole ligamentum flavum and separated the boundary between the dural sacs with a nerve stripping ion, which is same as the En bloc resection of the ligamentum flavum technique reported by Luis M reported (35). The ligamentum flavum was not removed until sufficient medial facetectomy was performed, as this approach shields the dura mater and nerve roots from duratomy or thermal injury.

It should be noted that the term “narrow spinal canal” was defined based on the distance from the spinous process root to the bilateral dorsal articular processes in the suprapedicle notch section CT image of the lumbar spinal stenosis segment, which is less than the working tubular diameter (1.6 cm) in our center. To avoid ambiguity, the lumbar degenerative lateral recess stenosis associated with a narrow spinal canal referred to in this article does not have broad clinical representation, and its indications are limited to spinous process hyperplasia, articular process aggregation, ligamentum flavum hypertrophy, and no acute disc herniation.

## Conclusion

According to our retrospective observation of 25 patients, combined with the findings of previous literature, the contralateral approach with a microscope and tubular retractor system for ipsilateral decompression in patients with lumbar lateral recess stenosis and a narrow spinal canal can reduce damage to the articular processes, which may be beneficial for postoperative stability of the lumbar spine. However, there are several limitations to this study. First, this study is a retrospective study of case series involving a small number of cases and having a short follow-up period, which prevented the detection of complications such as the development of chronic vertebral column instability and recurred lateral stenosis. Second, although we demonstrated better lateral recess decompression in our cases, but absence of a comparable control group within the same study, this is another limitation of our study. Third, measurement of the reduction change may be inaccurate in reflecting the width of the bilateral articular facet and bilateral articular facet change in the suprapedicle notch section on CT scan with bias. A further follow-up evaluation with multicenter, large-scale randomized clinical trials would be necessary to prove the efficacy of contralateral approach in the long term.

## Data availability statement

The raw data supporting the conclusions of this article will be made available by the authors, without undue reservation.

## Ethics statement

The studies involving human participants were reviewed and approved by Ethics Committee of Wuxi Clinical College of Anhui Medical University. Written informed consent to participate in this study was provided by the participants’ legal guardian/next of kin. The studies were conducted in accordance with the local legislation and institutional requirements. The participants provided their written informed consent to participate in this study.

## Author contributions

LS: Writing – original draft, Investigation. QM: Project administration, Writing – review & editing, Visualization. FD: Writing – original draft, Project administration, Data curation. WZ: Writing – review & editing, Resources, Project administration, Methodology. MS: Writing – review & editing. YW: Writing – review & editing, Visualization, Supervision, Conceptualization.
